# Clinical research updates

**DOI:** 10.1111/camh.12739

**Published:** 2024-10-24

**Authors:** Marinos Kyriakopoulos, Polyvios Christofi, Katerina Tselika, Asimina Paleologou

**Affiliations:** ^1^ First Department of Psychiatry National and Kapodistrian University of Athens Athens Greece; ^2^ Department of Child and Adolescent Psychiatry, Institute of Psychiatry Psychology and Neuroscience King's College London London UK; ^3^ South London and Maudsley NHS Foundation Trust London UK

## Early environmental predictors for attention‐deficit hyperactivity disorder, autism spectrum disorder and their co‐occurrence

Polyvios Christofi

National and Kapodistrian University of Athens

Attention‐deficit hyperactivity disorder (ADHD) and Autism Spectrum Disorder (ASD) are prevalent, lifelong neurodevelopmental disorders associated with significant behavioural, academic, emotional, and adaptive challenges. These conditions are more common in males, exhibit high heritability, and have a complex aetiology. Furthermore, they frequently co‐occur with estimates of 30–50% of individuals with ASD having ADHD symptoms, and two‐thirds of individuals with ADHD having ASD symptoms. This suggests shared developmental pathways and risk factors that have not been adequately explored as most research addresses these conditions separately.

Lebeña and colleagues (2024) aimed to identify pre‐ and perinatal risk factors and, for the first time, consider early environmental psychosocial exposures associated with ADHD, ASD, and their co‐occurrence. Data were collected from the ABIS‐study (All Babies in Southeast Sweden) cohort, which included 16,365 children born between 1997 and 1999, with a 22‐year follow‐up period. Diagnoses were obtained from the Swedish National Diagnosis Register. The study found cumulative incidences of 4.6% for ADHD, 1.7% for ASD and 1.1% for their co‐occurrence. Being male was a significant predictor for all three outcomes with a male‐to‐female ratio of 2:1 for both ADHD and ASD, compared to 3:1 reported in previous studies. ADHD showed significant associations with a family history of autoimmune diseases, preterm birth, lower birth weight, younger parental age, low‐parental education, single‐parent households, low‐household income, maternal smoking and tobacco exposure at one year of age. There were significant associations with serious life events during pregnancy, and shorter breastfeeding duration compared to both control and ASD groups. Statistically independent ADHD predictors were being male, lower household income, lower maternal education, younger mother, maternal smoking, serious life events during pregnancy, lower paternal education and short breastfeeding duration. ASD was associated with maternal disease, infections during pregnancy, vaginal delivery compared to caesarean section, maternal age over 36 years, low‐maternal education and low‐household income. Statistically independent ASD predictors were being male, lower household income, and lower maternal education together with paternal Swedish nationality. The co‐occurrence group was associated with higher prevalence of autoimmune diseases in the family, preterm births, low‐parental education, single parent, low‐household income, lack of support during pregnancy, lack of security for mother and child, maternal smoking and serious life events at 1 year of age. Statistically independent predictors of the co‐occurrence were being male, lower household income and family history of autoimmune disorders.

The study's strengths included its large, population‐based sample and long follow‐up period, which enabled robust statistical analysis. Limitations included the lack of data on family history, which might have provided additional insights into genetic influences. The reliance on parental reports for some data may have introduced recall bias, though this was mitigated by cross‐referencing with medical records. In conclusion, the study suggests that ADHD, ASD, and their co‐occurrence have distinct but overlapping aetiologies, with ADHD possibly being more influenced by environmental psychosocial factors than ASD. The findings highlighted the importance of early‐life interventions such as supporting families facing socioeconomic challenges to potentially reduce the burden of these disorders.

**Reference**

Lebeña, A., Faresjö, Å., Jones, M.P., Bengtsson, F., Faresjö, T., Ludvigsson, J. (2024). Early environmental predictors for attention‐deficit hyperactivity disorder (ADHD), autism spectrum disorder (ASD) and their co‐occurrence: The prospective ABIS‐study. *Science Reports* 14, 14759.

## Using machine‐learning methods to identify early‐life predictors of 11‐year language outcome

Katerina Tselika

National and Kapodistrian University of Athens

Being able to identify early in life children who are at risk of persistent language disorders at a later age is of major importance, because early speech and language intervention may positively contribute to their later sociobehavioural, academic, emotional and quality‐of‐life outcomes.

Gasparini and colleagues (2023) aimed to identify preschool indicators that predict language outcomes in late childhood. They used the early Language Victoria Study (ELVS), a longitudinal cohort study (*n* = 1910) tracking language and communication from infancy to adolescence. Children were recruited from 8‐months old, from different socioeconomic status strata of Melbourne, Victoria, through the Maternal and Child Health Service, hearing screening visits and press advertising. The sample excluded children who were diagnosed at 8‐months old as having a disability, which would probably interfere later with their language development and parents whose level of English language was not sufficient to complete the questionnaires. Children had been assessed in 11 waves (ages 8 months to 13 years), and waves 1–4 were used to identify language predictors in the current study (ages 8, 12, 24 and 36 months). Different questionnaires for each age, regarding children's behaviour, symbolic play, language usage, home and family environment and parental stress, were employed. At age 11, 839 children had been retained and assessed for their language skills using the Clinical Evaluation of Language Fundamentals, Fourth Edition, Australian version (CELF‐4), a standardized test for identifying children with low‐language abilities. SuperLearrner algorithm was also used to estimate the accuracy of the selected set of predictors on language outcomes at that age.

The analysis had 1990 variables, 182 at 8 months, 644 at 12 months, 916 at 24 months and 248 at 36 months. A random forest, tree‐based machine‐learning method, separately for each collection wave, was run to estimate the importance of the variables. At 24 months, seven predictors related to vocabulary, symbolic play, pragmatics and behaviour yielded 73% sensitivity and 77% specificity for predicting low‐language abilities at 11 years. At 36 months, 7 predictors relating to morphosyntax, vocabulary, parent – child interactions, and parental stress yielded 75% sensitivity and 85% specificity for the same outcome. No measures with satisfactory accuracy at 8 and 12 months were identified. The importance of this investigation lies in the fact that two short sets of questions, answered by parents in less than a minute, when their children are at a very young age may accurately predict language abilities in late childhood.

The study has many strengths including the applicability of results, the fast administration of the questionnaire, the robust sampling methodology, the large cohort of participants and the possibility of replication in a different cohort. Limitations include the low‐retention rate of participants and the possible inherent subjectivity in using parent‐reported questionnaires.

**Reference**

Gasparini, L., Shepherd, D. A., Bavin, E. L., Eadie, P., Reilly, S., Morgan, A. T., & Wake, M. (2023). Using machine‐learning methods to identify early‐life predictors of 11‐year language outcome. *Journal of Child Psychology and Psychiatry*, 64, 1242–1252.

## Cognitive functioning in children and adolescents with depression: A systematic review and meta‐analysis

Asimina Paleologou

National and Kapodistrian University of Athens

Depression is a prevalent mental disorder among children and adolescents. Better understanding of the relationship between depression and cognitive difficulties is important for the prevention, management and long‐term outcomes of individuals affected by these conditions. Most studies focusing on this association concern the adult population.

Schumacher and colleagues (2024) conducted a systematic review and meta‐analysis, which aimed to provide an overview of the existing literature regarding cognitive functioning in depressed children and adolescents. The study followed strict guidelines, including the Joanna Briggs Institute Systematic Review Guidelines and the Preferred Reporting Items for Systematic Reviews and Meta‐Analyses (PRISMA) checklist. It included only paediatric subjects (up to 18 years old) with a current diagnosis of clinical depression according to standardized and validated diagnostic criteria (International Classification of Diseases, Diagnostic and Statistical Manual of Mental Disorders). Cognition was measured in one of five domains: memory, attention, executive function, language and processing speed.

The authors identified 17 studies that met the inclusion criteria, which were cross‐sectional (*n* = 15), longitudinal (*n* = 1) or interventional (*n* = 1) with a total number of 13,567 children (mean age 13.8 ± 2.2 years; 60% female). The majority of the studies focused on memory and attention. Children and adolescents with depression were found to exhibit lower scores on tests of cognitive functioning compared to control samples, specifically on tests of working memory, long‐term memory, attention, executive function and language. No differences were found in short‐term memory and in processing speed.

The strengths of this systematic review and meta‐analysis include its specific focus on children and adolescents and its robust study size and sample size. Limitations include the fact that cognitive domains were selected according to literature guidelines, which may lead to overlapping results between cognitive domains, the lack of commonly agreed cognitive tests across different studies and the exclusion of subjects with co‐morbid neurological conditions or intellectual disability.

In conclusion, this study highlighted that depressed children and adolescents are at risk for several cognitive impairments. Τhere is a need for better studying the link between depression and cognitive functioning in children and adolescents. Understanding their interconnection is likely to assist in the development of more effective treatments taking into account not only affective but also cognitive symptoms of depression.

**Reference**

Schumacher, A., Campisi, S.C., Khalfan, A.F., Merriman, K., Williams, T.C., Korczak, D.J (2024). Cognitive functioning in children and adolescents with depression: Α systematic review and meta‐analysis. *European Neuropsychopharmacology*, 79, 49–58.

## Ethics information

No ethical approval was required for these updates.

## Data Availability

Data sharing is not applicable to this article as no new data were created or analyzed in this study.

